# COVID-19 cases reported in Colorado following screening at selected US airports, January – July 2020

**DOI:** 10.1186/s13104-023-06339-6

**Published:** 2023-04-27

**Authors:** Anna Shaum, Tye Harlow, Reena K. Gulati, Andre Berro, Jennifer House

**Affiliations:** 1 Division of Global Migration and Quarantine, Centers for Disease Control and Prevention, Atlanta, USA; 2 Colorado Department of Public Health and Environment, Colorado, USA; 3 Bill & Melinda Gates Foundation, Seattle, USA

**Keywords:** SARS-CoV-2, COVID-19, Airports, Air travel, Borders

## Abstract

**Objective:**

We sought to estimate the proportion of air travelers who may have been infected with SARS-CoV-2 upon arrival to Colorado by comparing data on Colorado residents screened upon entering the US to COVID-19 cases reported in the state. Data on Colorado’s screened passengers arriving into the US between January 17 and July 30, 2020 were compared to Colorado’s Electronic Disease Reporting System. We conducted a descriptive analysis of true matches, including age, gender, case status, symptom status, time from arrival to symptom onset (days), and time from arrival to specimen collection date (days).

**Results:**

Fourteen confirmed COVID-19 cases in travelers who were diagnosed within 14 days after arriving in Colorado were matched to the 8,272 travelers who underwent screening at 15 designated airports with a recorded destination of Colorado, or 0.2%. Most (N = 13/14 or 93%) of these infected travelers arrived in Colorado in March 2020; 12 (86%) of them were symptomatic. Entry screening for COVID-19 and the sharing of traveler information with the Colorado Department of Public Health and Environment appeared to identify few cases early in the pandemic. Symptom-based entry screening and sharing of traveler information was minimally effective at decreasing travel-associated COVID-19 transmission.

## Introduction

Coronavirus disease 2019 (COVID-19), caused by severe acute respiratory syndrome coronavirus 2 (SARS-CoV-2), was first reported by China to the World Health Organization on December 31, 2019 [1]. On Jan 17, 2020, the US Centers for Disease Control and Prevention (CDC), with support from the Department of Homeland Security, began entry screening at select US airports of international air passengers arriving from areas determined to be high-risk due to widespread COVID-19 transmission. Shortly after, the outbreak was declared a public health emergency in the United States [2, 3]. By July 30, 2020, international air passengers originating from mainland China, Iran, the Schengen Zone (which includes much of Western Europe), Ireland, the United Kingdom, and Brazil were routed to 15 selected airports (termed “F15 airports”) across the US for entry screening [4]. Entry screening involved a temperature check, administering a questionnaire about COVID-19 symptoms, and observing passengers for any visible signs of illness [5]. Contact information for passengers screened at F15 airports, regardless of symptoms, was collected by CDC and provided to state health departments via secure electronic data sharing for follow-up at the states’ discretion. However, despite transmitting passenger information, states’ ability to conduct follow-up were affected by the accuracy and completeness of the data, availability of resources, and competing priorities in the context of the ongoing spread of COVID-19 in the US. Passenger information was transmitted to state health departments only for passengers from whom a US phone number and complete US address were obtained. Due to these issues and the choice of some states to not receive traveler information, CDC shared information on approximately 68% of passengers that underwent entry screening [5].

According to a CDC analysis, entry screening efforts identified few infected passengers. Of approximately 766,044 passengers screened between January 17, 2020, and September 13, 2020, when entry screening was discontinued, 9 (0.001%) were identified during the screening process and subsequently confirmed to have COVID-19 [5]. However, given the inability of symptom-based screening to identify asymptomatic or presymptomatic infected persons, or those incubating infection [6], the extent to which passengers cleared during screening at airports were infected or subsequently developed COVID-19 remains unknown. Answering this question is integral to evaluating the effectiveness of symptom-based screening at airports, and may inform future efforts for mitigating COVID-19 introduction through international arrivals at US ports of entry.

We sought to estimate the proportion of travelers who may have been incubating or had asymptomatic or presymptomatic infection upon arrival to Colorado by comparing data on Colorado residents screened through this program to COVID-19 cases reported in the state.

### Objectives

Our primary objective was to estimate the proportion of Colorado residents screened between January 17– July 30, 2020, at the F15 airports who were subsequently diagnosed with confirmed or probable COVID-19 within one incubation period (14 days) after arrival.

## Methods

Data on screened passengers with a recorded destination in Colorado from the CDC’s eManifest system—which recorded information on passengers screened at any of the F15 airports—were compared to Colorado’s Electronic Disease Reporting System (CEDRS), which contains all confirmed and probable COVID-19 cases in the State of Colorado. Suspect cases reported to the Colorado Department of Public Health and the Environment (CDPHE) are also entered into CEDRS (suspect cases are not a reportable condition). CEDRS also includes cases deleted as duplicates or as out-of-state transfers. We applied Council of State and Territorial Epidemiologists (CSTE) case definitions to categorize confirmed and probable cases [7]. CDPHE defined suspect cases as those meeting supportive laboratory evidence (positive serology) with no prior history of being a confirmed or probable case.

Data from eManifest and CEDRS were matched on first name, last name, date of birth, city, phone number, and address. Passengers were included if they arrived between Jan 17 – Jul 30, 2020.

**Fig. 1:**
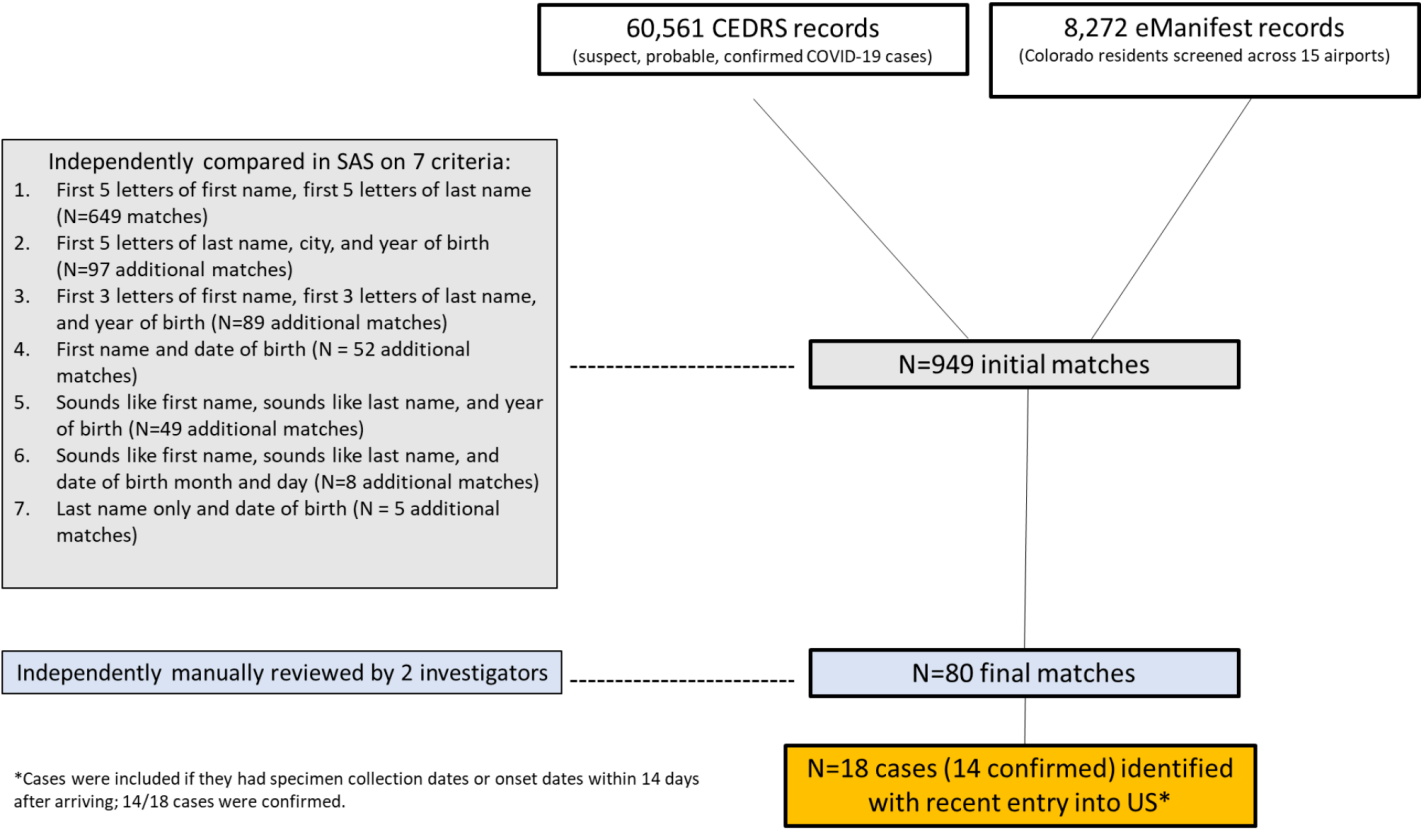
Process to identify matches between Colorado’s Electronic Disease Recording System (CEDRS) of COVID-19 cases and the Centers for Disease Control and Prevention’s eManifest system’s records of Colorado residents screened at US airports during the early months of the pandemic (January-July 2020)

Data for persons identified as having confirmed, probable, or suspect COVID-19 in the CEDRS database who matched a passenger identified in eManifest were used to create an analytic dataset comprising true matches. CEDRS includes both specimen collection date and symptom onset date, and in situations where both were available, the earlier of the dates was utilized as diagnosis date for all cases. Data were matched through a two-step process: first, CEDRS and eManifest records were compared electronically based on select criteria (Fig. 1.). Then, all resulting in potential electronic matches were independently reviewed by two investigators to confirm true matches. Discordant matches were excluded.

We conducted a descriptive analysis of true matches, including age, gender, case status, symptom status, time from arrival to symptom onset (days), and time from arrival to specimen collection date (days). Where data were available, we stratified data for persons who tested positive for SARS-CoV-2 by F15 airport. We analyzed data in SAS 9.4. The protocol received a non-research determination from CDC.

## Results

A total of 60,561 COVID-19 cases in CEDRS and 8,272 records from eManifest were identified from January 17 –July 31, 2020. Initial comparisons identified 949 potential matches based on select matching criteria using SAS (Fig. 1). A manual review of the potential 949 matches yielded 80 (8%) true matches.

Eighteen of the 80 (23%) travelers identified as true matches developed symptoms or had a positive laboratory test result for SARS-CoV-2 during the 14 days following their arrival into the United States, including 14 confirmed and 4 suspected (IgG+) case matches. The 14 travelers with confirmed COVID-19 represented 0.2% of the 8,272 screened travelers who arrived in Colorado during the analysis period. Most (N = 13/14) of these travelers arrived in Colorado in March 2020 (Table 1).

**Table 1:** Characteristics of international travelers diagnosed with COVID-19 in Colorado within 14 days after undergoing entry screening at US airports, January – July 2020

Age Group	(median = 51 years)	*n*		*%*
	18–30	4		29
	31–55	6		42
	56–75	4		29
**Gender**				
	Female	8		57
	Male	6		43
**Symptom Status**				
	Symptomatic	12		86
	Asymptomatic	1		7
	Missing	1		7
**Screening airport** ^**^				
	EWR	5		36
	ATL	4		29
	ORD	1		7
	LAX	1		7
	DFW	1		7
	IAD	1		7
	SEA	1		7

*N = 14; confirmed CO cases only. Suspect cases excluded.

** EWR = Newark Liberty International Airport; ATL = Hartsfield Jackson Airport; ORD = Chicago O’Hare Airport; LAX = Los Angeles International Airport; DFW = Dallas Fort Worth International Airport; IAD = Dulles International Airport; SEA = Seattle-Tacoma International Airport

Of the 14 travelers with confirmed COVID-19, 12 (86%) were symptomatic; 10 (71%) had symptom onset or specimen collection dates after arriving in the US, and 4 (29%) had symptom onset dates on the day of arrival (Table 2). Among those for whom symptom onset dates after arrival were available (N = 8), the median onset was three days (range = 2–5 days) after arrival. Among the 14 travelers with confirmed COVID-19, the median time from arrival to specimen collection date was six days (range = 1–57 days). Most were screened at Newark Liberty International Airport (N = 5) or Atlanta Hartsfield Jackson Airport (N = 4).

**Table 2:** Months of US entry for air passengers diagnosed with confirmed COVID-19 in Colorado within 14 days after undergoing entry screening* at one of 15 US airports, and all screened passengers destined for Colorado, January—July 2020

	2020							Total	
	January	February	March	April	May	June	July	**n**	**%**
No. with COVID-19^**^	0	0	13	0	0	1	0	14	0.2
No. Screened	3	223	3552	499	611	1293	2091	8272	100

*Countries of passenger origin designated for entry screening changed during the response. Screening was initiated on January 17, 2020, for passengers from Hubei Province, China. On February 3, screening was expanded to include mainland China; on Mar 2, Iran was included; on March 14, the Schengen zone was added, and on March 17, the United Kingdom and Ireland were included; on May 27, entry screening expanded further to include passengers arriving from Brazil.**Given the 14-day incubation period, to compare within each month, cases are sorted by the month of screening, not by the specimen collection or onset date

## Discussion

Screening of arriving international air passengers for symptoms of COVID-19 at major US airports during the first eight months of the pandemic, collecting their contact information, and subsequently transmitting their contact information to state health departments for follow-up, required substantial time and human resources [5]. Earlier evaluations determined that entry screening was ineffective for identifying travelers with laboratory-confirmed COVID-19 before entry into the US [5, 8]. Our results support this finding and highlight that few cases would have been identified from the international traveler data sent daily to CPDHE. Of the 8,272 Colorado residents screened from January to July 2020, less than 0.5% were matched with a confirmed COVID-19 case. A prospective study conducted by the California Department of Public Health early in the pandemic showed similar findings as only three (0.02%) COVID-19 cases were identified out of the 12,061 travelers for whom they were sent information. California discontinued using the traveler data sent by CDC on March 17, 2020 [8].

In our analysis, most travelers diagnosed with COVID-19 during the early months of the pandemic developed symptoms after arrival, highlighting the challenges of symptom-based entry screening. Additionally, screening did not include testing, which may have yielded different results.

Four travelers included in our analysis reported that their symptoms started on the day of arrival; however, these cases were not detected by the symptom-based screening process. This finding further highlights the difficulty of relying on symptom-based screening to detect COVID-19 which can have a mild or nonspecific clinical presentation [5, 9, 10]. Additionally, travelers may deny symptoms during the screening process or take steps to avoid detection, such as through use of medication to reduce fevers or suppress coughs [5].

## Conclusion

Sharing of air passenger information with CDPHE following entry screening in U.S. airports appeared to identify few COVID-19 cases early in the pandemic. Our analysis supports previous findings that showed symptom-based entry screening was minimally effective at decreasing travel-associated COVID-19 transmission because of the potential for travel during the incubation period or while infectious with presymptomatic or asymptomatic COVID-19 [5, 6, 8]. Subsequent strategies such as the requirement for international air passengers to provide proof of a negative test result or documentation of having recently recovered from COVID-19 before boarding a flight to the United States [11], mask requirements in transportation settings [12], and enhanced traveler education, may have been more effective in reducing further introduction of SARS-CoV-2 during travel.

## Limitations

Our analysis had at least five limitations. First, the information available in eManifest and CEDRS is subject to data quality limitations, as not all passengers who traveled to Colorado had or were willing to provide complete and accurate information (such as a US based phone number or address) available in the eManifest system. Only passengers—primarily US citizens or lawful permanent residents—in areas deemed to be high-risk were routed to the F15 airports [5], so persons entering the US from other countries that may have had COVID-19 infections early in the pandemic could have been missed. Furthermore, the eManifest data were collected manually, which may have led to transcription errors in specific fields, making matching by name challenging. In CEDRS, specific symptom information on cases was unavailable early in the pandemic and was missing for our identified cases and dates for symptom onset are subject to recall bias. Second, molecular testing for SARS-CoV-2 was not widely available in Colorado early in the response. Thus, it is possible that persons who traveled during the early stages of the pandemic and developed COVID-19 were never captured in CEDRS. Third, some cases may have been locally acquired after travel. Fourth, some infections, especially if asymptomatic, may have occurred prior to travel but not been detected at the time, with subsequent detection due to persistent shedding of viral RNA [13]. Fifth, Colorado’s surveillance systems may have missed infected travelers with mild or asymptomatic infections.

## Data Availability

The datasets used and/or analyzed during the current project may be available on reasonable request.

## Abbreviations

CDC: Centers for Disease Control and Prevention
CDPHE: Colorado Department of Public Health and Environment
CEDRS: Colorado’s Electronic Disease Reporting System
COVID-19: Coronavirus disease 2019
CSTE: Council of State and Territorial Epidemiologists
SARS-CoV-2: Severe acute respiratory syndrome coronavirus 2
